# Supplementary material for: Learning curve and surgical outcome of robotic assisted colorectal surgery with ERAS program
Source: Sci Rep. 2022 Nov 29;12:20566. doi: 10.1038/s41598-022-24665-w (PMC9709162; doi:10.1038/s41598-022-24665-w)
Supplement: Supplementary file 2 — Supplementary Information 2. [file 41598_2022_24665_MOESM2_ESM.zip › RAL Raw data-Ñ[▒K/108/OP2_ñw╝╨░O▒Kñσ.pdf]

正式摘要(電子簽章：000000224567873)

W52 / W52

臺中榮民總醫院

Operation Note

[redacted]

- 手術日期：2021/09/01
- 手術醫師：VS 林俊余，CR 陳璿夫，R 林柏志
- Anesthesia : GE
- Position : Supine
- Aseptic agents : Betadine Alcohol

Diagnosis

- Pre-operative diagnosis : S colon ca s/p AR with leak.
- Post-operative diagnosis : Ditto.s/p Hartmann's procedure(end D-colonostomy)

Surgical Indication

- for infection control and fecal diversion

Operation

- 1. Hartmann's procedure(end D-colostomy+ lower rectal stump).
  2. TA (60-4.8, X1) were applied for colon resection.
  3. End D-colostomy was created over the LLQ area
  4. Two 12 Fr. penrose drainage tube was inserted from RLQ site and it was placed over the pelvic area
  5. the fascia was also closed with retension Nylon

Finding

- 1. dirty ascites with fecal like material was presented after entering the abdominal cavity
  2. The small bowel was checked thoroughly and neither rupture site nor ischemia change was found
  3. After dissection of the adhesion part between the intestine and the pelvic wall, the leak site was found over the previous anastomosis line with a little hole about 0.5cm

Procedure

- 1. General endotracheal anesthesia was induced. Foley was further inserted. Then the patient was placed in the supine position. The abdomen was prepped and draped in standard sterile fashion.
  2. A lower vertical midline incision was made. This was deepened through the subcutaneous tissues and hemostasis was achieved with electrocautery.
  3. The abdomen was explored and wound retractor (Rogan) was set up.
  4. dirty ascites with fecal like content was noted.
  5. The small bowel was inspected and pulled out using a moist pad. No suspicious lesions nor ischemia change was noted.
  6. The dissection was performed until the lower rectal area was reached. And the leak side was found over the previous anastomosis line with a little hole about 0.5cm and TA 60-4.8 wass applied for resection. The D colon was resected.
  7. The possible bleeder was checked thoroughly and no obvious bleeding was noted from the retal stump.
  8. Two 12 Fr. penrose drainage tube was inserted from RLQ site and it was placed over the pelvic area
  9. The end-colostomy site was determined over the LLQ area. An oval incision was made and the incision was deepended through subcutaneous tissues, muscular and fascial layers until an opening

- sufficient for three fingers was created.
  10. The end of D-colon was then delivered through this opening without tension or torsion. It was fixed to the peritoneum medially with interrupted 3-0 mersilk and laterally to the peritoneum in such a manner as to eliminate the lateral trap.
  11. The fascia was closed with interrupted suture via totally interrupted mattress sutures via retension Nylon sutures
  12. The colostomy was then mature with multiple interrupted sutures via 3-O PDS.
  13. The patient tolerated the procedure well and was brought to the SICU under relative stable situation.
- Estimated blood loss: 500 c.c
- 器械數量: 正確
- 紗布數量: 正確

Image

- PAINT
  - abdomen

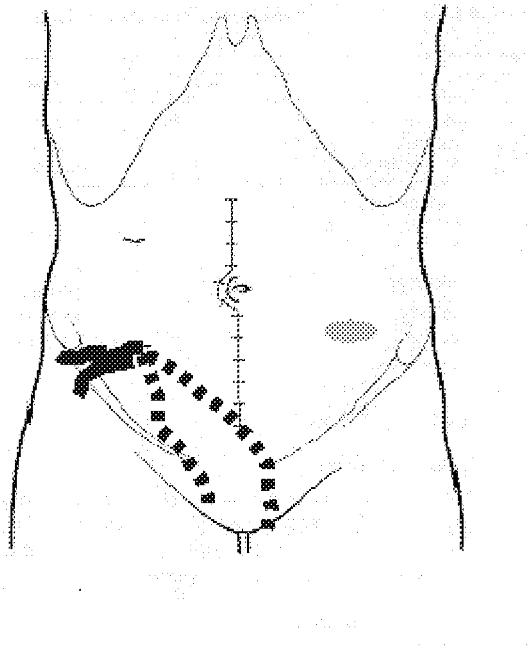

  - colon & rectum

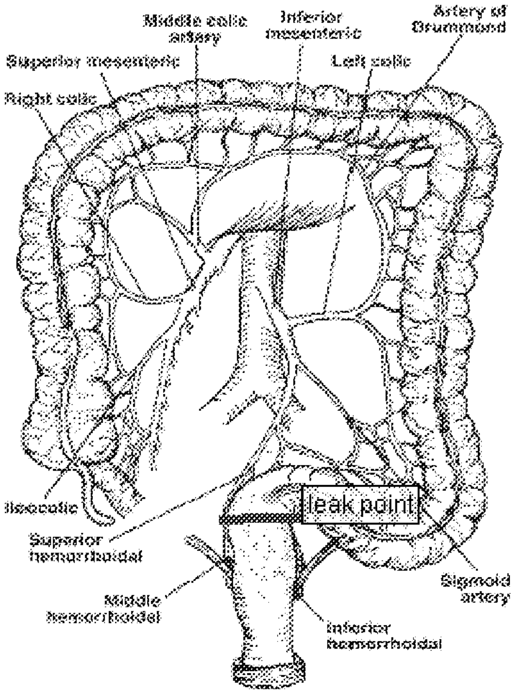

主治 2222H林俊余 / 醫師 2310K陳璿夫

正式摘要(**電子簽章：000000224567873)**

W52 / W52

doctorRank:B
